# Outcomes of oncoplastic breast surgery compared to breast-conserving surgery in breast cancer patients in a developing country: a retrospective cohort study

**DOI:** 10.12669/pjms.40.1.7360

**Published:** 2024

**Authors:** Lubna M. Vohra, Nifasat Farooqi, Uswa Jiwani, Danish Ali

**Affiliations:** 1 Lubna M. Vohra, FCPS, Department of Surgery, Aga Khan University Hospital Karachi, Pakistan; 2 Nifasat Farooqi, FCPS, Department of Surgery, Aga Khan University Hospital Karachi, Pakistan; 3 Uswa Jiwani, MBBS, Department of Surgery, Aga Khan University Hospital Karachi, Pakistan; 4 Danish Ali, MBBS, Department of Surgery, Aga Khan University Hospital Karachi, Pakistan

**Keywords:** Breast-conserving surgery, Oncoplastic surgery, Developing country, Re-excision rate

## Abstract

**Background and Objective:**

Breast-conserving surgery (BCS) with adjuvant radiotherapy remains the standard of care for early breast cancers in Pakistan. We sought to compare the outcomes of BCS with oncoplastic surgery (OPS), a relatively infrequent approach to breast cancer treatment in the country.

**Methods:**

This retrospective cohort study was conducted at Aga Khan University Hospital and Ziauddin Hospital in Karachi. Patients who had biopsy-proven Stage-I to III breast cancer and underwent either OPS or BCS between August 1, 2016, and December 31, 2021, were identified and followed for 30 days. Data were collected by reviewing patient files and electronic records.

**Results:**

A total of 481 patients were included in the study, where 204 (42.4%) underwent BCS and 277 (57.6%) underwent OPS. Mean tumor volume (146.8 vs. 90.4 cm3), and postoperative complications (2.2 % vs. 0%) were higher in OPS while the frequency of positive margins was greater in the BCS group (15.7 % vs. 2.2 %). There were no significant differences in the histologic type of tumor between the two groups.

**Conclusion:**

OPS is a valid alternative approach to breast cancer treatment that can be offered to women with early stage, locally advanced, multifocal or tumors at complex locations owing to the reduced occurrence of positive margins and thus lowered re-excision rates.

## INTRODUCTION

Breast-conserving surgery (BCS) with adjuvant radiotherapy has largely replaced mastectomy as the standard treatment for early breast cancers on grounds of breast preservation along with comparable oncological safety.^1^–^3^ However, larger tumors,^4^ a greater volume of resection, and medial tumor locations^5^ are some limitations to achieving good cosmetic outcomes with BCS. The need for favorable cosmesis without compromising oncological safety has paved the way for oncoplastic surgery (OPS) in breast cancer management. OPS is the integration of oncologic and plastic breast surgical techniques; making it an alternative treatment option for early, as well as larger breast tumors that would otherwise be treatable via mastectomy. OPS incorporates plastic surgery strategies such as decreasing scar visibility, volume displacement using mastopexy techniques, and volume replacement using autologous flaps or implants to achieve better cosmetic outcomes.^6^

Literature comparing OPS with standard BCS shows similar results in terms of local recurrence rate^7^,^8^ but with better cosmetic outcomes.^9^ While the Western world is working towards standardizing the approach^10^,^11^ OPS is still a new concept in Pakistan. There are only a few trained oncoplastic breast surgeons in the country and thus there is scarce data for OPS available from Pakistan.^12^,^13^ With this study, we sought to compare the surgical outcomes of OPS to those of BCS emphasizing the volume of tumor resected and margin positivity amongst patients with early-stage breast cancer in the Pakistani population. Therefore, the objectives of this study were to compare 30-day postoperative outcomes and complications (seroma development, fat necrosis, and wound infection) between the OPS and BCS groups and to delineate the surgical procedures carried out for OPS with differing tumor locations.

## METHODS

A multicenter retrospective cohort study was carried out at the Aga Khan University Hospital and Ziauddin Hospital in Karachi, Pakistan from August 1, 2016, to December 31, 2021, after obtaining an exemption (ERC# 2018-0349-258) from the Ethical Review Committees of the two institutes September 2018. The research activity was performed as per protocol and informed consent was not required (exemption from the Ethics Review Committee). Following the institutional data retention policies, all the data collected for this study had been stored on an encrypted drive for seven years which is accessible only by the research team. De-identified patient records were used during data entry to make sure that patient confidentiality was maintained throughout.

All female patients who presented with biopsy-proven American Joint Committee on Cancer (AJCC) 8^th^ Edition Stage I to III breast cancers and had undergone either OPS or BCT during the study period were included. Patients who had incomplete records or did not follow up at institutions (till 30 days postoperatively) were excluded from the analysis. A total of 481 patients were included in the study: 415 from Aga Khan University Hospital and 66 patients from Ziauddin Hospital. Demographic, perioperative, operative, and histopathologic data were collected retrospectively by reviewing patient files and electronic records.

A multidisciplinary team involving a breast surgeon, radiologist, and medical oncologist decided which patients would receive neoadjuvant chemotherapy to downsize the tumor. The type of surgery to be performed was determined by the primary surgeon with subsequent endorsement by the multidisciplinary team. OPS was considered in cases where there was a significant disparity in breast-to-tumor ratio or if the locations of tumors made it difficult to attain good cosmetic outcomes postoperatively with BCS. Patients were informed about the surgery and the expected outcomes, with the help of pictorial drawings, as well as the possibility of complications before undertaking adequate procedural consent. Tissue from the tumor bed was clipped in all patients scheduled for neoadjuvant chemotherapy for BCS. Post neoadjuvant ultrasound or mammographic guidance was used to localize the clip/residual tumor. All margins were marked before specimens were fixed in formalin and sent to the histopathology lab. Axillary lymph node dissection was added for patients with positive sentinel lymph nodes on biopsy. Specimens were analyzed by trained pathologists. Specimen volume was calculated by multiplying the length, breadth, and width. The maximum tumor dimension mentioned in the pathology report was used for this study. Six margins (anterior, posterior, medial, lateral, superior, and inferior) were assessed for tumor cells. Margins were considered negative if no tumor cells were found on the inked surface as per the SSO/ASTRO guidelines.^14^ All excised lymph nodes were evaluated for tumor metastasis. Patients were followed for 30 days to assess readmission due to postoperative complications. Seromas were diagnosed clinically by breast examination and confirmed by ultrasound. A single aspiration of seroma resulted in complete resolution in all cases. Wound infection was confirmed and treated based on culture and sensitivity.

Data were analyzed using the IBM Statistical Package for Social Sciences (SPSS) (Version 22). The mean was calculated for the quantitative variable and frequency and percentages were computed for categorical variables. The data were compared using the Chi-square test of independence/Fisher’s exact test (as appropriate) for categorical variables while the Mann-Whitney U test was used to test for relationships between the quantitative variable. A *p*-value of <0.05 was considered significant throughout.

## RESULTS

A total of 481 female patients were included in the analysis, with 277 (57.6%) patients in the OPS group and 204 (42.4%) in the BCS group. The median age of the entire cohort was 50 years, while those of the OPS and BCS groups were 49 years, and 51 years respectively, and the minor difference was not statistically significant *p*=0.513. Tumor bed characteristics were reported for patients with responses to neoadjuvant chemotherapy (Table-I). Neoadjuvant chemotherapy administration was significantly different between the two groups 39% of patients from the OPS group vs. 28.4% from the BCS group (p = 0.022). Invasive ductal carcinoma made up most of the tumors: 87.3% in the OPS group and 94.1% in the BCS group. The mean tumor volume between the two groups differed by 56.4 cm^3^ (148.8 cm^3^ in the OPS group vs. 90.4 cm^3^ in the BCS group) which was statistically significant, p < 0.001. Postoperative margin positivity was significantly higher in the BCS group 15.7% vs. 2.2% in the OPS group (p < 0.001) (Table-I).

**Table-I:** Tumor characteristics.

	n = 481		

Characteristics	OPS, n=277 (57.6)	BCS, n=204 (42.4)	p-value
***Neoadjuvant chemotherapy, n (%)***			0.022
Yes	108 (39.0)	58 (28.4)	
No	169 (61.0)	146 (71.6)	
***Histology, n (%)***			0.430
Invasive ductal carcinoma	242 (87.3)	192 (94.1)	
Invasive lobular carcinoma	06 (2.2)	00 (0.0)	
Metaplastic carcinoma	06 (2.2)	04 (2.0)	
Ductal carcinoma in-situ	13 (4.7)	06 (2.9)	
Others	10 (3.6)	02 (1.0)	
***AJCC 8^th^ Edition T-stage, n (%)***			
No tumor identified (post-NAC)	15 (10.3)	12 (10.8)	
T_1_	55 (37.7)	55 (49.5)	
T_2_	76 (52.1)	44 (39.6)	
T_3_	0	0	
***AJCC 8^th^ Edition N-stage, n (%)***			
N_0_	276 (87.1)	139 (84.7)	
N_1_	41 (12.9)	25 (15.3)	
Mean tumor volume, cm^3^	148.8	90.4	<0.001
***Margin positivity, n (%)***			
No	271 (97.8)	172 (84.3)	<0.001
Yes	6 (2.2)	32 (15.7)	

***Note:*** Bold p-values are significant at a 0.05 level, OPS Oncoplastic surgery, BCS Breast-conserving surgery, AJCC American Joint Committee on Cancer, NAC Neoadjuvant chemotherapy.

The most performed oncoplastic procedure was lateral mammoplasty (32.1%) followed by the round block technique (21.2%), and then the inverted T technique in 20.8% of cases (Table II). A total of 38 patients (7.9%) had positive margins on histopathological analysis of which 32 (15.7%) had undergone BCS, while others underwent OPS (2.17%) (*p <* 0.001). Seventeen patients from the BCS group (8.33%) underwent margin re-excision, and four of them showed the presence of residual tumor cells in the resected specimens. All patients had negative margins on re-excision. Out of six patients with positive margins from the OPS group, four (66.7%) patients underwent mastectomy while two (33.3%) did not undergo any further procedure. A total of six patients (2.16%) developed postoperative complications during the 30-day follow-up period, and all belonged to the OPS group (Table-III). None of these patients required re-admissions due to complications within the 30-day follow-up period.

**Table-II:** Tumor location and technique employed during oncoplastic surgery

	N = 277*		

Quadrant	Technique	n (%)	Complications
Tumors of the upper pole (11 to 1 o’clock)	Round block technique	72 (26%)	3
Tumors of the upper outer quadrant (1 to 3 o’clock)	Lateral mammoplasty	47 (16.9%)	0
	Rotation Flap	26 (9.3%)	0
Tumors of the lower pole (5 to 7 o’clock)	Inverted T technique	43 (15.5%)	0
	Reduction mammoplasty	14 (5.05%)	0
Tumors of the lower inner quadrant (7 to 9 o’clock)	Inverted T technique	11 (3.9%)	1
	Matrix rotation	26 (9.3%)	0
Tumors of the upper inner quadrant (9 to 11 o’clock)	Round block technique	25 (9.0%)	2
	Batwing	07 (2.5%)	0
Central	Grisotti	06 (2.2%)	0

***Note:***

* N denotes the number of patients undergoing OPF (277 out of 481).

**Table-III:** Postoperative complications

		N = 481		

Complications	OPS	BCS	Total	p-value
Seroma, n (%)	5 (3.4)	0 (0)	5 (1.9)	<0.001
Fat necrosis, n (%)	0 (0)	0 (0)	0 (0)	
Wound infection, n (%)	1 (0.7)	0 (0)	1 (0.4)	
Total	6 (4.1)	0 (0)	6 (2.3)	

***Note:*** Bold p-value is significant at a 0.05 level, OPS Oncoplastic surgery, BCS Breast-conserving surgery.

## DISCUSSION

It has been well-established that positive margins increase the odds of local recurrence of breast cancer.^15^ The results of this study showed that OPS when compared to BCS, had a lower frequency of positive margins and a greater volume of tissue resection, akin to patterns seen in the literature.^16^ A meta-analysis by Losken et al. demonstrated a significantly lower positive margin rate in the OPS group 12.3% vs. 20.6% in the BCS group.^17^ Identical to this study, Chauhan et al. found positive margins and tumor recurrence only in patients who underwent BCS.^18^

BCS has suboptimal cosmetic outcomes when more than 20% of the breast tissue is resected.^19^ The introduction of OPS has allowed for a greater amount of tissue resection without affecting cosmetic outcomes, thus potentially assisting in attaining wider margins. This approach has been extended to treat ‘extreme’ cases as described by Silverstein et al. Extreme oncoplasty includes tumors with positive lymph nodes; tumors that would likely need radiation even after a mastectomy; and tumors that are > 5 cm, multifocal, and multicentric^20^ – albeit with a higher percentage of re-excision.^21^ Whether OPS is used to address early or extreme breast cancer, the tumor volume is generally larger, as demonstrated in the literature^22^, also concurred by this study. The mean tumor volume amongst the OPS group was 58.4 cm^3^ greater (p < 0.001) than the BCS group in this study.

OPS is known to have a higher frequency of short-term complications, with the most reported rate being 20%.^23^ This may be due to the resection of a larger volume, longer operating time, and greater skill required to perform the procedure. This is the first study from Pakistan to compare the rates of short-term complications between OPS and BCS. Our study showed a higher percentage of overall complications in the OPS (2.1%) compared to the BCS group (0%), but none were significant enough to require hospitalization. Barring major procedures, the higher rate of complications in OPS does not usually delay adjuvant therapy.^24^ Wound seroma was the most common early post-surgery complication; hence, we learnt that in larger dissections an operative drain placement can avoid such an occurrence.

A systematic review by De La Cruz et al. reported a weighted average re-excision rate of 6% after OPS and concluded that these rates were lower than those for BCS.^25^ Literature from Pakistan showed varying rates of re-excision after OPS surgeries 9.5% and 1.6% by Qureshi et al. and Abidi et al. respectively; the latter also mentioned that re-excision rates with BCS were 9.8% in the study population 12,13. In this study, 44.7% (17 out of 38 patients with positive margins) did not follow up for adequate margin re-excision; thus, the rates of margin positivity were used as a proxy for margin re-excision in this study. Margin positivity was seen in 15.7% of patients with BCS vs. 2.17% in the OPS group. While the results of this study showed a similar pattern to De La Cruz et al. and Abidi et al. there is an absolute difference in the incidence of re-excision.^13^,^25^ Despite the higher rate of re-excision after BCS, studies have shown no significant difference in time to adjuvant therapy between BCS and OPS.^26^,^27^ However, a low rate of re-excision is still preferred as subsequent surgeries can worsen cosmetic outcomes, cause patient distress, and increase cost and morbidity.

During this study half of the patients with positive margins did not appear for re-excision; the team could reach only two patients who responded to having pursued mastectomy at another hospital. The main reason for this was speculated to be the high out-of-pocket cost of treatment at private tertiary care hospitals. As this study was carried out at two private hospitals in a large metropolitan city of Pakistan it may not be representative of patients from other parts of the country. This study did not evaluate the postoperative cosmetic outcomes as patient records indicated only subjective satisfaction or dissatisfaction with the cosmetic outcome.

OPS is still in its early stages in Pakistan^28^ and studies from Pakistan have shown that improved patient satisfaction and surgical outcomes.^13^,^29^,^30^ With the advent of accredited breast surgery fellowship programs in Pakistan, it can be predicted that the growing interest in breast surgery research will allow for more meaningful and patient-oriented research in the field of OPS and hence, the outcomes.^31^

Based on the results of these findings, OPS appears to be the better surgical treatment option for candidate patients as it may lower the rate of positive margins, and hence the re-excision rate. However, further studies are warranted to determine the risk of overtreatment with OPS, and its short- and long-term oncologic safety compared to that of the current standard treatment of early breast cancer in the population of interest. We recommend that the use of OPS should be studied for smaller lesions; if it lowers the rates of re-excision, it can lower the economic burden and overcome the cultural norms in developing countries.

## CONCLUSION

Oncoplastic surgery shows beneficial oncologic safety compared to breast-conserving surgery for early stage, locally advanced and multifocal breast cancers or tumors at complex locations. It allows for a greater volume of excision and therefore, can be used to treat larger breast tumors in complex locations. However, further prospective studies on this topic are warranted to determine the efficacy and benefits of this approach in our population.

### Authors Contribution:

**LV:** Conceived, designed and did manuscript writing, is responsible for integrity of research.

**NF**, **UJ**, & **DA:** Did data collection and manuscript writing.

**LV:** Did review and final approval of manuscript.
